# Pre- and peri-hematopoietic cell transplant management of disseminated non-*Helicobacter pylori Helicobacter* infection in X-linked agammaglobulinemia: Case series and literature review

**DOI:** 10.1016/j.clim.2026.110685

**Published:** 2026-02-17

**Authors:** Rachel Lee, Danielle E. Arnold, Mark Parta, Sung-Yun Pai, John P. Dekker, Sanchita Das, Rose Lee, Jennifer Cuellar-Rodriguez, Jenna Bergerson, Mary M. Czech

**Affiliations:** a Laboratory of Clinical Immunology and Microbiology, National Institute of Allergy and Infectious Diseases, National Institutes of Health, Bethesda, MD, United States of America; b Immune Deficiency Cellular Therapy Program, Center for Cancer Research, National Cancer Institute, National Institutes of Health, Bethesda, MD, USA; c Clinical Research Directorate/Clinical Monitoring Research Program, Frederick National Laboratory for Cancer Research Sponsored by the National Cancer Institute, Bethesda, MD, United States of America; d Department of Laboratory Medicine, National Institutes of Health, Bethesda, MD, USA

**Keywords:** X-linked agammaglobulinemia (XLA), Allogeneic hematopoietic cell transplant (HCT), *Helicobacter bilis*, *Helicobacter cinaedi*, Non-*Helicobacter pylori Helicobacter* infection, Chronic cellulitis, Osteomyelitis

## Abstract

Non-*Helicobacter pylori Helicobacter* (NHPH) species are recognized as a cause of chronic systemic infection, cellulitis, and osteomyelitis in patients with X-linked agammaglobulinemia (XLA). Diagnosis and treatment are challenging due to fastidious growth, lack of standardized therapies, and frequent recurrence. We describe two cases of disseminated NHPH infection in XLA, including a patient where allogeneic hematopoietic cell transplant (HCT) was incorporated into management to achieve durable immune reconstitution and cure infection. One patient with disseminated *Helicobacter bilis* osteomyelitis discontinued antibacterials 14 months post-HCT following immune reconstitution. Another patient with disseminated *Helicobacter cinaedi* remains on antibacterials and is being evaluated for HCT. In select cases, HCT may represent a potential option to correct the immunodeficiency and enable infection clearance, with antibacterial therapy continued through HCT until systemic immunosuppression is withdrawn, immune reconstitution is documented, and infection resolves.

## 1. Introduction

X-linked agammaglobulinemia (XLA) is a primary immunodeficiency characterized by mutations of the Bruton’s tyrosine kinase (BTK) gene on the X-chromosome, leading to absent mature B cells and markedly reduced levels of all immunoglobulin isotypes. Patients present with recurrent sinopulmonary bacterial infections that can progress to chronic lung disease, as well as persistent bacterial or viral gastrointestinal infections that can disseminate [1–3]. Immunoglobulin replacement therapy (IgRT) has significantly improved clinical outcomes by restoring IgG levels. However, patients remain vulnerable to enteric pathogens because IgA deficiency, which compromises mucosal immunity, is not corrected by IgRT. As a result, enteric organisms such as non-*Helicobacter pylori Helicobacter* (NHPH) species, can cross the gastrointestinal barrier and cause disseminated infection [4]. Diagnosis and treatment of disseminated NHPH infections in XLA patients are complicated by the organism’s fastidious growth requirements, absence of standardized therapies, and high risk of persistence or recurrence in the setting of immunodeficiency [4]. As with other inborn errors of immunity, allogeneic hematopoietic cell transplant (HCT) may be the only curative intervention for an infectious complication. Previous reports suggest that this may also be the case for XLA patients with refractory infections [5–11]. However, there are no data regarding the feasibility of HCT as a curative treatment of NHPH infections in XLA.

We present two cases of disseminated NHPH infection in patients with XLA. One patient has received HCT with resolution of infection, demonstrating that durable immune reconstitution through HCT may lead to definitive cure. The second patient is undergoing evaluation for HCT due to persistent infection. These cases suggest that HCT could represent a potential therapeutic option for XLA patients with refractory NHPH infection.

### Case 1. Chronic disseminated Helicobacter bilis with osteomyelitis in a patient with XLA.

A 17-year-old man from the United States with XLA (*BTK* c.46C > T, p.Gln16Ter) presented for management of disseminated *Helicobacter bilis* infection. He was diagnosed with XLA at one year of age and maintained on IgRT. His history included recurrent sinopulmonary infections and biopsy-proven nodular regenerative hyperplasia (NRH) of the liver.

**Fig. 1.**
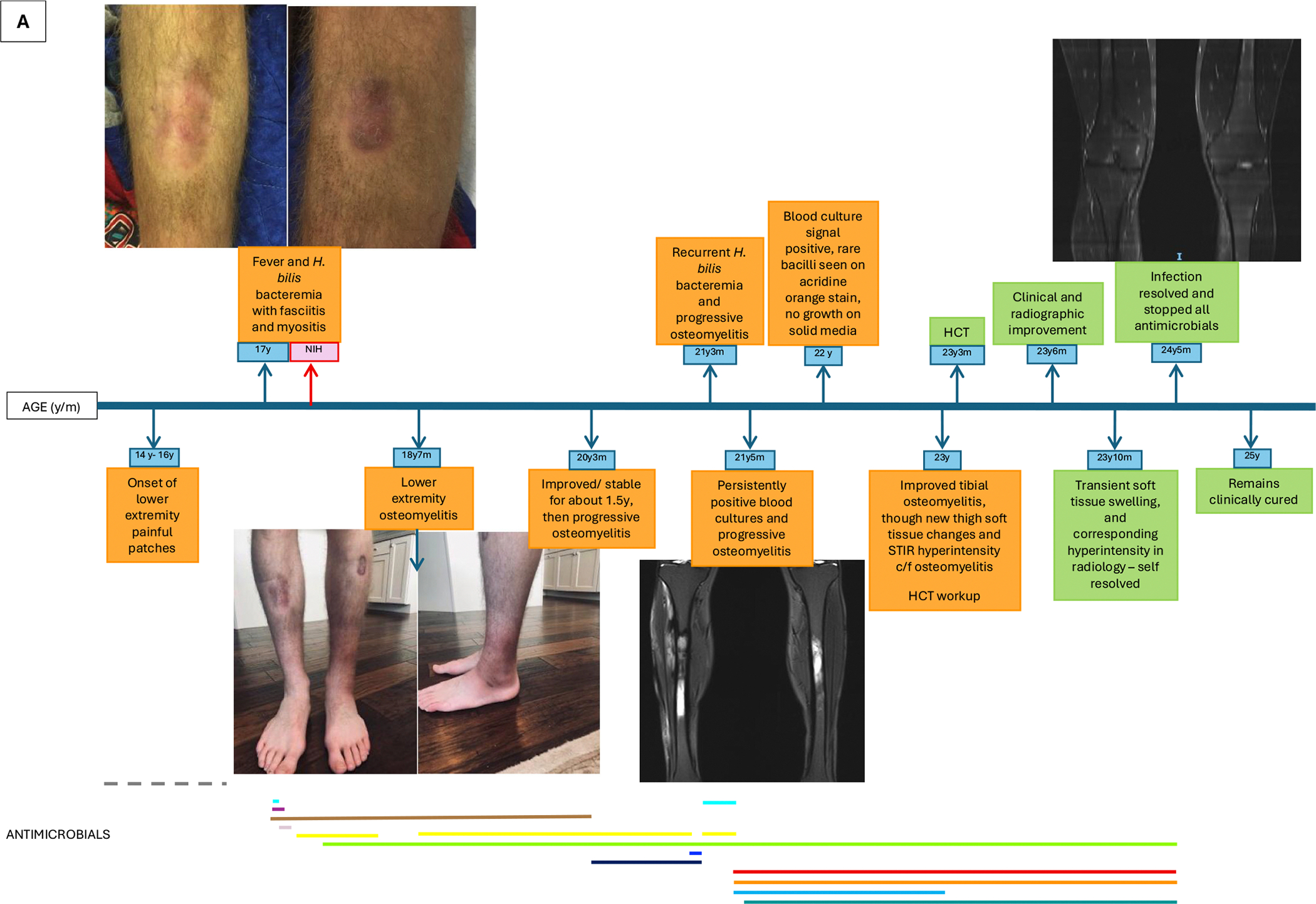

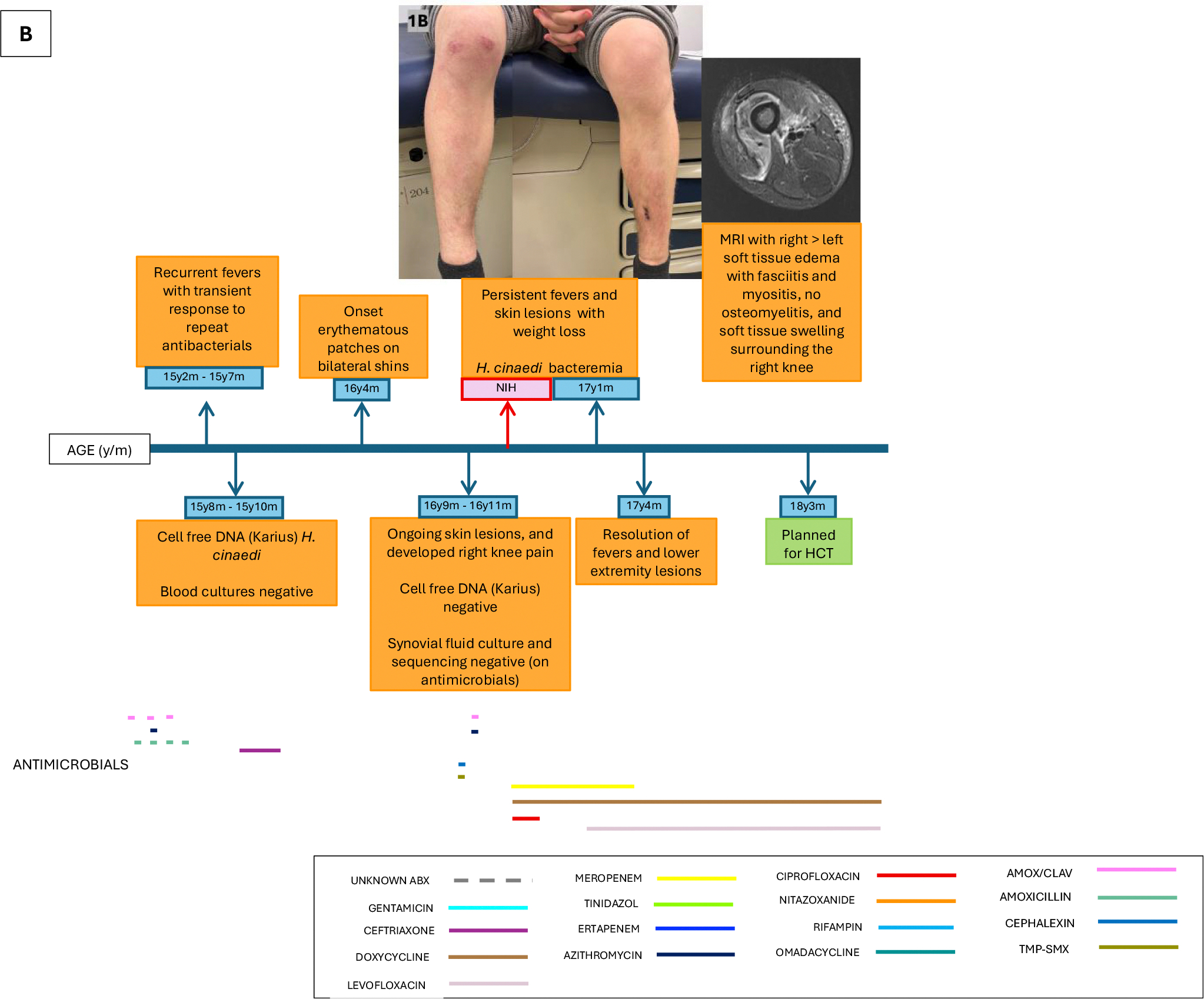
Timeline of infection and antimicrobial therapy in XLA patient with disseminated *H. bilis* infection (A) and patient with disseminated *H. cinaedi* infection (B).

The complex course of his *Helicobacter* infection is summarized in Fig. 1A. At age 14, he developed an erythematous patch on his right shin, with involvement of the contralateral leg appearing the following year. By age 16, he experienced worsening erythema, swelling, and pain in the lower extremities, most prominent in the left ankle, and later accompanied by fevers following methotrexate administered for presumed localized scleroderma. Antimicrobial regimens administered during this time at outside institutions are not known. At age 17, MRI demonstrated soft tissue edema, and skin biopsy of the left ankle showed chronic inflammatory changes. Retrospective pathology review showed filamentous bacteria in the subcutis highlighted by Steiner stain, which is an argyrophilic silver impregnation method that can facilitate visualization of thin, poorly staining bacteria such as *Helicobacter* species. Plasma cell-free DNA sequencing (Karius^®^, Redwood City, CA) detected *Helicobacter* DNA fragments, and blood cultures confirmed *Helicobacter bilis* (susceptibilities in Table 1). The patient was initiated on multiple antibacterial regimens, inclusive of ceftriaxone, gentamicin, doxycycline, and levofloxacin, and subsequently referred to our center.

Despite more than 40 days of antibacterials, including doxycycline and levofloxacin for which there were low minimum inhibitory concentrations (MICs), blood cultures remained positive (growth at 12 days), with stool cultures also yielding *H. bilis*. Repeat blood cultures demonstrated increased MICs compared to the blood isolate 43 days prior (Table 1). The susceptibility for the *H. bilis* in the blood and stool were similar, except for discordance of the meropenem MIC (≤0.06 in blood vs 2 in stool). He was treated with meropenem and doxycycline, with the subsequent addition of tinidazole, with clinical improvement.

After one year of therapy, meropenem was temporarily discontinued for three months, leading to recurrent symptoms within six weeks of cessation, characterized by night sweats, ankle pain, and new imaging evidence of osteomyelitis. Meropenem was restarted with subsequent clinical and radiographic improvement. At age 20, however, he developed new sites of osteomyelitis in the left lower extremity, prompting a switch from doxycycline to azithromycin, while meropenem and tedizolid were continued. Approximately 11 months later, meropenem was replaced with ertapenem for ease of administration, but this was followed by recrudescent *Helicobacter* bacteremia (growth at 9 days), rising inflammatory markers, and new right tibial osteomyelitis. Meropenem was restarted and gentamicin was added. Two months later, he developed gentamicin-induced ototoxicity, with *H. bilis* bacteremia still detectable (growth at 16 days), and radiographic progression of left tibial osteomyelitis. He was transitioned to an all-oral regimen of ciprofloxacin, tinidazole, nitazoxanide, rifampin, and the later addition of omadacycline. Twenty-two days later, lower extremity erythema resolved, and blood cultures cleared. Tibial osteomyelitis radiographically improved, though new bilateral thigh soft tissue abnormalities and femoral STIR hyperintensity were detected.

At age 23, given persistent infection refractory to prolonged multidrug therapy in the context of XLA, he underwent allogeneic HCT to correct the immunodeficiency and achieve infection clearance. His antimicrobial regimen leading up to HCT included ciprofloxacin, tinidazole, nitazoxanide, omadacycline, and rifampin. Rifampin was discontinued pre-HCT due to anticipated drug-drug interactions, and the other four antimicrobials were continued through transplant. He received a reduced intensity conditioning matched sibling donor bone marrow graft from his sister, who was not a carrier of the BTK variant. He did not have any flare of *Helicobacter* or other infectious complications in the early post-HCT period. At 7 months post-HCT, MRI of the lower extremities showed non-specific subcutaneous edema, but no new bone changes, which self-resolved. Monthly IgRT was successfully discontinued at one-year post-HCT. Follow-up imaging at 14 months showed no active infection. CD19+ cell count at that time was normal with emerging class-switched memory B cells (pre- and post-HCT lymphocyte subsets and immunoglobulin levels are presented in Supplemental Table 1). All antibiotics were discontinued 14 months post-HCT, following seven years of consecutive administration. He remains clinically well and infection-free 21 months post-HCT, and 7 months post antimicrobial discontinuation.

### Case 2. Chronic disseminated Helicobacter cinaedi in a patient with XLA.

A 17-year-old man from the United States with XLA (*BTK* c.1844 1851del, p.Arg615GlnfsTer19) was referred to our hospital for management of suspected disseminated *Helicobacter cinaedi* infection. He was diagnosed with XLA at 2 years of age and maintained on IgRT. He had a history of recurrent rhinosinusitis, gastroenteritis, and biopsy- proven NRH of the liver.

The course of his *Helicobacter* infection is summarized in Fig. 1B. Beginning at age 15, he developed recurrent fevers that transiently improved with empiric antibiotics. Plasma cell-free DNA sequencing (Karius^®^, Redwood City, CA) identified *H. cinaedi*, although blood cultures were negative. He completed six weeks of ceftriaxone with resolution of fevers. At age 16, he developed painful, erythematous, flat patches on the bilateral shins, and subsequent right knee pain with a non-diagnostic work up. He received several courses of empiric antibacterials, during which time repeat plasma cell-free DNA sequencing was negative for *Helicobacter*. The lower extremity erythematous patches persisted, and he later developed daily fevers with a 10-pound unintentional weight loss over one month.

At age 17, he presented to our center. MRI of the lower extremities showed soft tissue edema, fasciitis, and myositis, without osteomyelitis. Blood cultures obtained during this evaluation grew *H. cinaedi* at two days (susceptibilities in Table 1). He was treated with meropenem, ciprofloxacin, and doxycycline, with rapid defervescence. Ciprofloxacin was held for one month, and then switched to levofloxacin due to concern for thrombocytopenia. Three months following the start of targeted antibacterial therapy, he had resolution of the lower extremity lesions, normalization of inflammatory markers, and clearance of blood cultures. Meropenem was discontinued, and he remains on levofloxacin and doxycycline. He is undergoing evaluation for HCT, with ongoing antibacterial therapy planned through the post-HCT period.

## 2. Discussion

Our case series illustrates the unique vulnerability of patients with XLA to disseminated NHPH infections and provides insights into diagnostic and management considerations surrounding HCT. One patient with disseminated *Helicobacter bilis* complicated by osteomyelitis discontinued antibacterials 14 months post-HCT following immune reconstitution. Another patient with disseminated *Helicobacter cinaedi* remains on antibacterials as a bridge to potential HCT and immune reconstitution.

### 2.1. Clinical presentation

Infections caused by enterohepatic NHPH species are recognized in individuals with XLA. The most clinically relevant are *H. bilis* and *H. cinaedi*, although other species have also been reported. Clinical presentation typically begins with cellulitic lesions that appear as erythematous areas and gradually evolve over months to years into painful patches. Chronic cellulitis is often accompanied by fevers and bacteremia. The lower extremities are more frequently involved than the upper extremities, possibly due to more frequent microtrauma in the lower extremities that disrupts local barriers and greater blood flow demands that may facilitate hematogenous seeding. In some cases, cellulitis progresses to osteomyelitis or pyoderma gangrenosum-like ulcers [4,12,13]. Therefore, in patients with XLA who present with chronic or recurrent cellulitis or osteomyelitis, clinicians should maintain a high index of suspicion for disseminated NHPH infection.

### 2.2. Diagnosis

Diagnosing disseminated *Helicobacter* is challenging due to their fastidious nature. Most patients with disseminated NHPH are diagnosed by blood cultures and/or 16S sequencing identification of bacteria in a skin biopsy [4]. We recommend both diagnostics when feasible.

At least three sets of blood cultures should be obtained at different time points to increase sensitivity with more blood volume and to account for intermittent low-level bacteremia. Blood cultures should be obtained in standard aerobic bottles and incubated in automated systems. Because prior antibiotic exposure may sterilize cultures, samples are ideally collected off antibacterials. Growth can be slow, with cultures often requiring more than 5 days of incubation [14]. In our first patient with *H. bilis* bacteremia, blood cultures became positive after a range of 9–16 days, whereas in the second patient with *H. cinaedi* bacteremia, blood cultures were positive at 2 days. At our center, blood cultures are incubated for 21 days when *Helicobacter* is suspected. Even when blood cultures flag positive in automated systems, organisms may not be visible on gram stain due to faint staining or atypical morphology, potentially leading to misinterpretation as a false positive flagging. To improve detection when pre-test probability is high, we recommend a acridine orange stain [15] and/or a carbol fuschin counterstain instead of safranin for gram stains. Carbol fuchsin-enhanced gram stains have consistently demonstrated greater sensitive than culture for detecting *Campylobacter* spp. from direct stool smears [16,17], and given their similar morphology, *Helicobacter* can be visualized in a comparable manner.

When positive blood culture bottles are plated, several media can support growth [18]; we preferentially use brain heart infusion agar. Plates should be incubated under microaerophilic conditions with supplemental hydrogen, ideally using a gas mixture of 6% O_2_, 7% H_2_, 7% CO_2_, and 80% N_2_ [18], which approximates the gastrointestinal environment where *Helicobacter* typically resides.

Identification remains challenging. MALDI-TOF identification can be limited by incomplete spectral representation of NHPH organisms and is highly dependent on the specific database in use [19]. Consequently, 16S sequencing is often necessary for definitive species-level identification. However, even 16S sequencing may not reliably distinguish *Helicobacter* spp. due to the acquisition of large intervening sequences (IVS) within the 16S rRNA gene during adaptation and coevolution of *Helicobacter* strains in hosts [20].

Biopsy specimens should be collected for microbiologic and pathologic evaluation. Specimens for microbiology should be collected in sterile containers without preservatives. They should be transported to the microbiology laboratory as soon as possible to be plated and intubated under microaerophilic conditions with supplemental hydrogen. Plates should undergo prolonged incubation, and identification should proceed as outlined for blood cultures. Pathology evaluation should include stains that would highlight *Helicobacter*, such as Warthin-Starry, Giemsa, Steiner stains, or *Helicobacter* immunohistochemistry.

Finally, our two cases, along with another report [12], highlight the potential role of plasma metagenomic next-generation sequencing in diagnosing disseminated NHPH, although this approach is constrained by cost, limited availability, and the inability to provide antibiotic MIC data.

### 2.3. Treatment

Therapy for disseminated NHPH infections is particularly challenging due to the absence of robust antimicrobial susceptibility profiles and clinical data. There are no standardized susceptibility breakpoints for NHPH. Clinical and Laboratory Standards Institute only provides breakpoints for *H. pylori* in relation to clarithromycin, while EUCAST provides breakpoints for a more expanded panel of antibacterials against *H. pylori* (Table 1). Therefore, providers confronted with treating NHPH infections must use a combination of available in vitro MIC testing, information derivative from *H. pylori*, and clinical reports treating NHPH infection.

There are currently no substantial published data describing MIC patterns for *H. bilis*. In vitro MIC data for *H. cinaedi* are derived primarily from studies of Japanese isolates where these infections appear more prevalent. Across these reports – covering a total of 113 *H. cinaedi* isolates with variable antibiotic testing - agar dilution demonstrated low MICs for imipenem (MIC_90_ range 0.125–0.25 mg/mL), tetracycline (MIC_90_ 1 mg/mL), and gentamicin (MIC_90_ range 0.5–1 mg/mL); a wider range for amoxicillin (MIC_90_ range 4–16 mg/mL); and high MICs for clarithromycin (MIC_90_ range 128- > 128 mg/mL) and ciprofloxacin (MIC_90_ 64–128 mg/mL) [21–23]. Notably, in our second patient, the *H. cinaedi* blood isolate showed low MICs for amoxicillin, clarithromycin, and fluoroquinolones, suggesting that published in vitro MIC profiles may not be generalizable across geographic regions, where patterns of antimicrobial use and resistance differ among clinical isolates. Whenever NHPH is recovered in culture, we recommend pursuing antibacterial MIC testing at a laboratory experienced with *Helicobacter*.

Treatment strategies for NHPH infections are extrapolated from those used for *H. pylori*. In the United States, current *H. pylori* treatment guidelines recommend combination regimens incorporating first-line agents such as tetracycline, amoxicillin, rifabutin, clarithromycin, and a nitroimidazole (either metronidazole or tinidazole) [24]. Both of our patients were treated with second generation tetracyclines (doxycycline), with our first patient subsequently receiving a third-generation tetracycline (omadacycline) due to its strong in vitro activity against *H. pylori*, including tetracycline-resistant strains [25]. Both patients were treated with beta-lactams, and the first, more treatment-experienced patient additionally received a rifamycin, macrolide, and nitroimidazole. Randomized controlled trials comparing metronidazole versus tinidazole have shown similar eradication rates for *H. pylori* [26,27], although one international registry study reported higher cure rates with tinidazole [28]. Tinidazole, although more expensive, is associated with fewer gastrointestinal side effects. Our first patient also received nitazoxanide, a broad-spectrum thiazolide antimicrobial effective against *H. pylori* [29]. Notably, nitazoxanide does not exhibit cross-resistance to metronidazole [30].

Case reports describing treatment of NHPH infection, particularly in XLA patients, are exceedingly limited compared with the literature on *H. pylori* therapy. Most published cases in XLA patients describe the use of a beta-lactam, typically a carbapenem, combined with an aminoglycoside either as primary therapy or as induction prior to transition to oral agents [4,12]. Both of our patients were treated with carbapenem, and our first patient also received a course of gentamicin. However, prolonged use of carbapenems and aminoglycosides is constrained by their need for intravenous administration, and aminoglycosides carry additional risks of nephrotoxicity and ototoxicity, the latter of which occurred in our patient.

Ultimately because antibacterial therapy for NHPH is non-standardized, patients are often treated with multidrug regimens [4]. Empiric therapy should consider a patient’s prior antibiotic exposures, particularly monotherapies that may have selected for resistance. When available, MIC testing may help guide therapy, with additional consideration for disease severity and sites – for example, select agents with good bone penetration for osteomyelitis. Even when MIC data are obtained, maintaining at least a two-drug regimen may reduce the risk of developing on-therapy resistance (as reported in a prior case of prolonged *H. cinaedi* treatment [31]), address potential infection with a heterogeneous *Helicobacter* population, tackle challenges in susceptibility testing, and mitigate the limited correlation between MIC profiles and clinical outcomes.

XLA patients with NHPH infections are often treated on the order of months to years [4]. Despite prolonged treatment, these infections are frequently refractory, with progression during targeted therapy or recrudescence occurring when antibacterial therapy is stopped. This was exemplified by both patients, with the first ultimately undergoing HCT to achieve definite cure for his chronic infection. To our knowledge, this is the first report of HCT being performed in XLA for NHPH infection. The rationale stems from the underlying immune defect, which hinders effective clearance and long-term infection control, and from the potential of HCT to restore functional immunity.

Historically, HCT in XLA has been described as a strategy to reduce dependence on IgRT, particularly in the setting of limited access or high cost [3,32,33]. More recently, allogeneic HCT has been proposed, and highlighted via case reports, as a curative option for XLA patients with refractory or life-threatening infections where immune reconstitution is required for cure. Nishimura et al. [6] provided a comprehensive review of 16 patients with XLA who underwent HCT for infection. Since that publication, one additional report has described an XLA patient who received HCT for infection [11]. Collectively, among these 17 patients, 11 patients underwent HCT for bacterial infections, three for chronic Aichi virus, two for chronic norovirus, and one for concurrent chronic *Campylobacter jejuni* and norovirus. Specific bacterial pathogens were not detailed in most cases, and none indicated a diagnosis of NHPH infection. Two patients died shortly following HCT – one from viral infection and one from Enterobacterales infection in the setting of gastrointestinal graft-versus-host disease. Excluding these early deaths, all others demonstrated improved infectious outcomes or cure.

In XLA patients with refractory disseminated NHPH infection – defined as progression during targeted therapy or recrudescence following cessation of antibacterial therapy - HCT may warrant consideration as part of the treatment strategy given that such infections are typically difficult to eradicate without correction of the underlying immunodeficiency. This recommendation is tempered by the inherent limitations of a single-patient experience, which may limit generalizability. Moreover, the substantial risks associated with allogeneic HCT - including infection, graft-versus-host disease, and treatment-related toxicities - must be carefully weighed against the potential benefit in treating refractory infection.

The optimal timing of HCT in the setting of refractory NHPH infection remains undefined. However, earlier consideration, prior to cumulative antimicrobial toxicity and/or irreversible end-organ damage may confer greater benefit with lower peri-transplant risk. From an infectious diseases perspective, we would consider XLA patients with disseminated NHPH infection as appropriate HCT candidates once blood cultures for *Helicobacter* have cleared and there is clinical improvement with declining inflammatory markers and/or documented radiographic improvement. We propose antibacterial therapy directed against NHPH should be continued through HCT and discontinued post-HCT when the following criteria are fulfilled: clinical, microbiologic, and radiographic resolution of infection; laboratory evidence of immune reconstitution of the deficient immune compartments; and discontinuation of all systemic immunosuppression. This approach aims to minimize the risk of relapse during the period of immune recovery.

## 3. Conclusion

These cases underscore the diagnostic and therapeutic complexities of managing chronic NHPH infections in patients with XLA. HCT may offer cure by correcting the underlying immunodeficiency. Emerging data support its role in select patients with life-threatening or refractory infections, including those with disseminated NHPH infection.

## Supplementary Material

MMC1

## Figures and Tables

**Table 1:** *Helicobacter* minimum inhibitory concentration (MIC) data for Case 1 and Case 2, and established *Helicobacter pylori* breakpoints.

	MICs (mcg/mL)								
	
	Case 1 – *Helicobacter bili* isolates overtime			Case 2 *-Helicobacter cinaedi* isolate	*Helicobacter pylori* – established breakpoints for reference				
			
	Blood cx at 17y, 3 m	Blood cx at 17y, 4 m	Stool cx at 17y, 4 m	Blood cx at age 17y, 1 m	CLSI			EUCAST	
						
					S	I	R	S ≤	R >

Amoxicillin				≤0.008				0.125	0.125
Ciprofloxacin	0.06	2	>4	≤0.03					
Levofloxacin				0.06				1	1
Clarithromycin	0.25	2	2	0.016	≤0.25	0.5	≥ 1.0	0.25	0.25
Doxycycline	≤0.12	1	1						
Erythromycin	≤0.12	>32	>32						
Meropenem	0.12	≤0.06	2						
Minocycline	≤0.25	≤0.25	≤0.25						
Tetracycline	0.25	8	8	≤0.03				1	1
Gentamicin				0.5					
Metronidazole								8	8
Rifampicin								1	1

All sensitivity testing for *H. bilis* isolates performed by Centers for Disease Control and Prevention, Atlanta, GA, USA.

For case 1, no sensitivity testing available for *Helicobacter bilis* blood culture isolate at 21y, 5 m.

Susceptibility testing for *H. cinaedi* performed by ARUP Laboratories, Salt Lake City, UT, USA.

CLSI – Clinical and Laboratory Standards Institute.

EUCAST – European Committee on Antimicrobial Susceptibility Testing.

S – sensitive; I – intermediate; R – resistant.

## Data Availability

No data was used for the research described in the article.

## Appendix A. Supplementary data

Supplementary data to this article can be found online at https://doi.org/10.1016/j.clim.2026.110685.

## Abbreviations:

HCT: hematopoietic cell transplant
IgRT: immunoglobulin replacement therapy
MIC: minimum inhibitory concentration
NHPH: non-*Helicobacter pylori Helicobacter*
XLA: X-linked agammaglobulinemia
