# Supplementary material for: Pre- and peri-hematopoietic cell transplant management of disseminated non-Helicobacter pylori Helicobacter infection in X-linked agammaglobulinemia: Case series and literature review
Source: Clin Immunol. Author manuscript; Available in PMC 2026 Mar 23. (PMC13006914; doi:10.1016/j.clim.2026.110685)
Supplement: MMC1 [file NIHMS2150458-supplement-MMC1.docx]

**Supplemental Table 1 – Patient 1 lymphocyte subsets and immunoglobulin levels pre- and post-HCT**

|  | **Pre-HCT** | **14 months post-HCT** |
| --- | --- | --- |
| **CD19+** | 0/mcL | 154/mcL |
| **CD3+** | 830/mcL | 138/mcL |
| **CD4+** | 516/mcL | 85/mcL |
| **CD8+** | 248/mcL | 47/mcL |
| **NK** | 153/mcL | 89/mcL |
| **IgG** | 714 mg/dL (on IgRT) | 758 mg/dL (off IgRT) |
| **IgA** | <5 mg/dL | <5 mg/dL |
| **IgM** | <5 mg/dL | 78 mg/dL |
